# Severe Myelotoxicity Associated with Thiopurine S-Methyltransferase*3A/*3C Polymorphisms in a Patient with Pediatric Leukemia and the Effect of Steroid Therapy

**DOI:** 10.4274/tjh.2013.0082

**Published:** 2014-12-05

**Authors:** Burcu Fatma Belen, Türkiz Gürsel, Nalan Akyürek, Meryem Albayrak, Zühre Kaya, Ülker Koçak

**Affiliations:** 1 Gazi University Faculty of Medicine, Department of Pediatric Hematology, Ankara, Turkey; 2 Gazi University Faculty of Medicine, Department of Pathology, Ankara, Turkey; 3 Kırıkkale University Faculty of Medicine, Department of Pediatric Hematology, Ankara, Turkey

**Keywords:** Myelosuppression, Thiopurine S-methyl transferase, Acute leukemia

## Abstract

Myelosuppression is a serious complication during treatment of acute lymphoblastic leukemia and the duration of myelosuppression is affected by underlying bone marrow failure syndromes and drug pharmacogenetics caused by genetic polymorphisms. Mutations in the thiopurine S-methyltransferase (TPMT) gene causing excessive myelosuppression during 6-mercaptopurine (MP) therapy may cause excessive bone marrow toxicity. We report the case of a 15-year-old girl with T-ALL who developed severe pancytopenia during consolidation and maintenance therapy despite reduction of the dose of MP to 5% of the standard dose. Prednisolone therapy produced a remarkable but transient bone marrow recovery. Analysis of common TPMT polymorphisms revealed TPMT *3A/*3C.

## Abstract (tr)

Miyelosupresyon, akut lenfoblastik lösemi tedavisinde görülen ciddi bir komplikasyon olup, miyelosupresyon süresi kemik iliği yetmezlik sendromlarından veya genetik polimorfizmin yol açtığı ilaç farmakogenetiğinden etkilenmektedir. Merkaptopürin metabolizasında yer alan Thiopurin s-metil transferaz (TPMT) enziminin azalmış aktivitesine neden olan polimorfizmleri artmış kemik iliği toksisitesine yol açar. Burada, 15 yaşında TPMT *3A/*3C ve MTHFR polimorfizmleri saptanan ve konsolidasyon/idame tedavisi boyunca MP’nin standart dozun %5’inde yoğun miyelosupresyon görülen ve steroid tedavisi ile geçici düzelme saptanan bir T-ALL olgusu sunulmaktadır.

## INTRODUCTION

Myelosuppression is a serious complication of chemotherapy in children with acute lymphoblastic leukemia (ALL). The duration and the severity of myelosuppression vary with genetic polymorphisms affecting drug pharmacokinetics and bone marrow failure syndromes [1,2]. The thiopurine S-methyl transferase (TPMT) enzyme is involved in the metabolism of 6-mercaptopurine (MP), a widely used cytostatic agent in childhood ALL. The levels of active MP metabolites are elevated during MP therapy in patients who carry TPMT mutations with decreased enzyme activity such as TPMT *2 (G238C), TPMT *3A (G460A and A719G), and TPMT *3C (A719G), leading to excessive bone marrow toxicity and being more profound in those with 2 as compared to 1 nonfunctional allele [2,3,4,5,6]. The contribution of methylenetetrahydrofolate reductase (MTHFR) mutations in MP-induced myelosuppression has not been well established [7,8]. We here describe the case of a 15-year-old girl with ALL and TPMT *3A/*3C and MTHFR polymorphisms who suffered from severe bone marrow suppression persisting during the consolidation and maintenance therapy.

## CASE PRESENTATION

**Figure 1:**
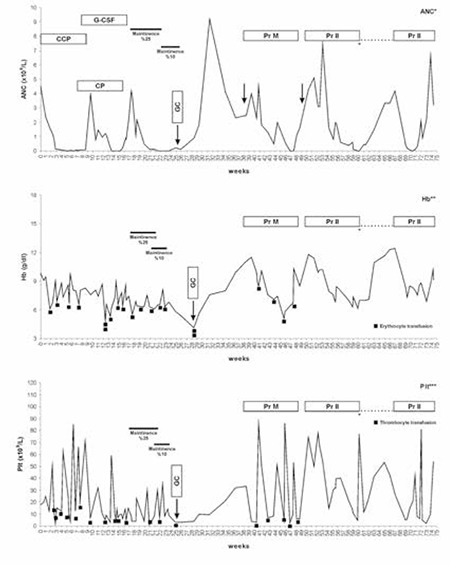
Absolute neutrophil counts, hemoglobin levels, and platelet counts during ALL BFM-95 chemotherapy. ANC: absolute neutrophil count, Hb: hemoglobin, Plt: platelet count, CCP: cytosine arabinoside + 6-mercaptopurine treatment, CP: cyclophosphamide, G-CSF: granulocyte colony-stimulating factor, GC: glucocorticoid (prednisolone), Pr M: Protocol M-BFM 95, Pr II: Protocol II-BFM-95. Blanks at the right end of the figure represent chemotherapy cessation due to hyperglycemia and hypertriglyceridemia during Protocol II.

A 15-year-old girl presented with a white blood cell (WBC) count of 150x10^9^/L, anemia, thrombocytopenia, and mediastinal enlargement. A diagnosis of T-cell ALL was made based on peripheral blood morphology and immunophenotyping by flow cytometer. Karyotyping disclosed t(11;14), but polymerase chain reaction (PCR) tests for t(4;11) and t(9;21) were negative. She was treated with the Berlin-Frankfurt-Münster (BFM) ALL-95 chemotherapy protocol [9]. She received induction chemotherapy uneventfully and achieved complete remission on day 33 with full bone marrow recovery. Five days after receiving the first 5 days of Protocol Ib, which contained 6-MP at 60 mg/m^2^ per day orally, cytosine arabinoside (c-ARA) at 75 mg/m^2^/day for 4 doses, and a single dose of 1000 mg/m^2^ cyclophosphamide, her WBC count, absolute neutrophil count (ANC), platelet count, and hemoglobin (Hb) level began to decrease and reached a minimum at week 14 (WBC: 0.8x10^9^/L, ANC :0x10^9^/L; platelets: 3x10^9^/L; Hb: 5 g/dL). Chemotherapy was stopped; granulocyte colony-stimulating factor (G-CSF) was started at 5 µg/kg/day and increased to 10 µg/kg/day. Six weeks later, WBC and ANC returned to normal but Hb level and platelet count remained low. Bone marrow aspirations yielded dry taps. TPMT genotyping for G238C, G460A, and A719G was reported as negative. As WBC and ANC were within normal limits, chemotherapy was restarted, but her counts rapidly dropped (Figure 1). She remained pancytopenic for a further 6-week period, during which chemotherapy was stopped, and numerous platelet and erythrocyte transfusions were given. Bone marrow biopsy showed cellularity of 5% with no residual leukemia and/or fibrosis. PCR analysis for parvovirus B19, Epstein-Barr virus, and cytomegalovirus and the diepoxybutane (DEB) test were negative. Because of protracted pancytopenia, the rest of Protocol Ib and intensification blocks were omitted and maintenance therapy with oral 6-MP and methotrexate (MTX) was started at 25% of protocol doses. Absolute neutrophil count dropped and remained below 0.8x10^9^/mm^3^ despite G-CSF therapy. Genotyping for MTHFR revealed heterozygousity for C677T and A1298C polymorphisms. We were informed that the patient had TPMT *3A/*3C polymorphisms; the previous report of wild-type TPMT was a transcription error. The doses of 6-MP and MTX were reduced to 10% of protocol doses, but the patient remained severely pancytopenic and transfusion-dependent. Bone marrow biopsy showed profound hypocellularity with occasional hematopoietic hot points and abundant fat cells, resembling aplastic anemia. Chemotherapy was stopped and oral prednisolone was started at a dose of 60 mg/m^2^ per day. WBC and platelet counts increased gradually starting from the second week, and bone marrow aspirate became normocellular and free of leukemic blasts at the end of 30 days of steroid therapy. Bone marrow biopsy showed 40% cellularity without residual blasts. As blood counts returned to normal limits, intensification therapy with 4 blocks of MTX at 5 g/m^2^/day and 6-MP at 2.5 mg/m^2^/day (5% of protocol dose) was given over the next 54 days. During this period, WBC count and ANC remained between 2 and 3x10^9^/L and platelet counts could be kept above 10x10^9^/L by weekly transfusions (Figure 1). MTX levels were calculated at 24, 36, 42, 48, and 54 h after infusion of MTX as below the highest upper limit to give additional folinic acid rescue. Reinduction therapy with vincristine, dexamethasone, L-asparaginase, and doxorubicine caused profound pancytopenia complicated with prolonged febrile episodes, severe hyperglycemia, and liver dysfunction. Therefore, the second part of the reinduction therapy was omitted, and maintenance therapy was restarted at 10% of standard doses. She completed the 5-week maintenance therapy with 6-MP and MTX doses ranging from 5% to 10% and from 8% to 16%, respectively. After termination of maintenance chemotherapy, blood counts gradually increased and reached normal range within 3 months. Informed consent was obtained.

## DISCUSSION AND REVIEW OF THE LITERATURE

Following intensive chemotherapy, blood counts recover within 2 to 4 weeks in most cases of ALL. Longer periods of bone marrow suppression are rare and can be caused by abnormalities of drug disposition pathways or congenital bone marrow failure syndromes with myelodysplasia. A clear relation with excessive bone marrow toxicity has been established only for thiopurine drugs and TPMT polymorphisms [1,2,3,4,5,6,10]. Patients homozygous or compound heterozygous for a nonfunctional TPMT allele develop pancytopenia 2 to 4 weeks after starting oral 6-MP and recover within 2-6 weeks [11,12]. The longest duration of pancytopenia was 137 days in an 8-year-old boy with ALL who was homozygous for TPMT *3A/*3A [13].

Bone marrow suppression in the present case with TPMT *3A/*3C polymorphisms was more severe as compared to previously described patients with 2 nonfunctional TPMT alleles. Pancytopenia developed shortly after introduction of 6-MP and persisted during maintenance therapy, despite reducing both 6-MP and MTX doses to 5% to 10%. Interruption of chemotherapy for 6-8 weeks and G-CSF therapy resulted in ANC recovery initially without significant increase in Hb levels or platelet counts, but the duration of bone marrow suppression was prolonged as 6-MP exposures increased, suggesting that myelotoxicity is dose-dependent. However, we did not investigate additional single nucleotide polymorphisms involved in thiopurine metabolism, such as inosine triphosphate pyrophosphatase, that might have been responsible for extreme bone marrow suppression in our patient [14]. She also had MTHFR C677T and A1298C polymorphisms, which cause reduced MTHFR activity, leading to decreased production of S-adenosylmethionine, a protector of the TPMT enzyme [15]. Karas-Kuzelicki et al. reported increased myelotoxicity in children with ALL who were heterozygous for at least one low-activity TPMT allele and for C677T and/or A1298C polymorphisms in the MTHFR gene [7]. In contrast, another study found lower frequency of severe myelotoxicity in pediatric ALL patients having both TPMT and C677T polymorphisms [8]. Interestingly, we did not observe excessive hematological toxicity during intensification therapy with high-dose (5 g/m^2^) MTX combined with low-dose MP (2.5% of protocol dose) in our patient, suggesting that compound heterozygosity for C677T and A1298C is not an additional risk factor for hematotoxicity in children with TPMT polymorphisms. An interesting finding in the present case is a quick bone marrow recovery induced by glucocorticoid therapy. Bone marrow cellularity increased from 5% at the beginning to 45% at the end of 4 weeks of 2 mg/kg prednisolone administration. Although steroid therapy is effective in the treatment of immune-mediated bone marrow suppression, its role in chemotherapy-induced bone marrow hypoplasia has not been well studied. A short course of high-dose methylprednisolone therapy was reported to increase CD34 (+) progenitor cells and shorten chemotherapy-induced neutropenia in children with leukemia [16,17]. The molecular mechanisms of steroid effects on bone marrow regeneration need to be illuminated.

Children with Fanconi anemia (FA) may experience severe bone marrow toxicity with alkylating agents like cyclophosphamide and busulphan [2,18]. Cyclophosphamide causes chromosomal breaks in FA patients. A recent study showed that cyclophosphamide-specific interstrand DNA cross-links were increased 15-fold in FA patients compared to non-FA patients [19]. Our patient had received high-dose cyclophosphamide in addition to 6-MP one week before development of initial bone marrow hypoplasia. She had none of the main clinical or laboratory features of FA, such as congenital defects, developmental abnormalities, elevated HbF level, or increased chromosomal fragility. Patients with myelodysplasia also show delayed bone marrow recovery after chemotherapy. Several cases of T-cell ALL have been described in patients with myelodysplasia associated with germline RUNX-1 mutation or microdeletion of 21q22 resulting in RUNX-1 deficiency [20]. The conventional karyotyping performed for our patient is not able to detect such a small deletion, but absence of thrombocytopenia history before development of leukemia and rapid bone marrow regeneration without dysplastic changes and complete hematologic reconstitution at day 33 of remission induction therapy exclude this possibility. Patients who are compound heterozygous for TPMT *3A/3C may have severe bone marrow hypoplasia even with minimal amounts of MP. Further investigation of other genetic factors involved in MP metabolism and measurement of the intracellular levels of thioguanine nucleotides may help in better treatment of chemotherapy-induced myelosuppression.

## CONCLUSION

Compound heterozygosity for TPMT *3A/3C may be associated with severe bone marrow hypoplasia, even with minimal amounts of MP, in children with ALL. The role of glucocorticoids on bone marrow regeneration after chemotherapy should be investigated further.

**Conflict of Interest Statement**

The authors of this paper have no conflicts of interest, including specific financial interests, relationships, and/or affiliations relevant to the subject matter or materials included.
